# Helminth mediated modulation of the systemic and mycobacterial antigen – stimulated cytokine profiles in extra-pulmonary tuberculosis

**DOI:** 10.1371/journal.pntd.0007265

**Published:** 2019-03-21

**Authors:** Gokul Raj Kathamuthu, Saravanan Munisankar, Rathinam Sridhar, Dhanaraj Baskaran, Subash Babu

**Affiliations:** 1 National Institutes of Health-NIRT-International Center for Excellence in Research, Chennai, India; 2 National Institute for Research in Tuberculosis (NIRT), Chennai, India; 3 Government Stanley Medical Hospital, Chennai, India; 4 Laboratory of Parasitic Diseases, National Institute of Allergy and Infectious Diseases, National Institutes of Health, Bethesda, Maryland, United States of America; Universidade Federal de Minas Gerais, BRAZIL

## Abstract

**Background:**

Helminth infections are known to regulate cytokine responses in both pulmonary and latent tuberculosis infection. Whether helminth infections also modulate cytokine responses in extra-pulmonary tuberculosis, specifically tuberculous lymphadenitis (TBL), has not been examined thus far.

**Methodology:**

Hence, to determine the cytokine profile in helminth-TBL coinfection, we measured the systemic and mycobacterial (TB)–antigen stimulated levels of Type 1, Type 2, Type 17, regulatory and pro-inflammatory cytokines in TBL individuals coinfected with or without *Strongyloides stercoralis* (Ss) infection.

**Significant findings:**

TBL-Ss+ individuals have significantly higher bacterial burdens in the affected lymph nodes in comparison to TBL-Ss- individuals. TBL-Ss+ individuals exhibit significantly enhanced plasma levels of Type 2 (IL-5 and IL-13), Type 17 (IL-17 and IL-22) and regulatory (IL-10) cytokines in comparison to TBL-Ss- individuals. In contrast, TBL-Ss+ individuals exhibit significantly diminished plasma levels of pro-inflammatory cytokines (IL-1α and GM-CSF) in comparison to TBL-Ss- individuals. TBL-Ss+ individuals also exhibit significantly diminished unstimulated or mycobacterial—antigen stimulated levels of Type 1, Type 17 or IL-1 family cytokines in comparison to TBL-Ss- individuals but no differences in mitogen stimulated cytokine levels.

**Conclusion:**

Therefore, our data reveal a profound influence of Ss infection on the bacteriological profile of TBL and suggesting that the underlying modulation of cytokine responses might be a mechanism by which this helminth infection could impart a detrimental effect on the pathogenesis of TBL disease.

## Author summary

*Strongyloides stercoralis* (Ss) infects about 30–100 million people worldwide and it is the main causative agent of strongyloidiasis, a chronic parasitic infection. Similarly, tuberculosis (TB) affects nearly 2 billion people and both Ss and TB are co-endemic as well as share a major global disease burden. Earlier studies have revealed that helminth co-infection can modify the effect produced by the host immunity against *Mycobacterium tuberculosis*. However, no studies have examined the association of helminth co-infection with extra-pulmonary TB, especially in tuberculous lymphadenitis (TBL). In the present study, we have addressed this gap by measuring the cytokine profile in co-infected helminth-TBL individuals. Our study first shows that TBL-Ss+ co-infection is associated with “higher bacterial burden” in the affected lymph nodes. Our study also shows that co-infection is associated with alterations in plasma cytokines, specifically Type 2, regulatory and Type 17 cytokines were elevated and pro-inflammatory cytokines were reduced. Our study demonstrates significantly diminished unstimulated or TB—antigen stimulated levels of Type 1, Type 17 or IL-1 family of cytokines in TBL-Ss+ coinfected individuals. Hence, we conclude that helminth coinfection can possibly skew the protective immunity in TBL disease or infection.

## Introduction

Helminth infections and tuberculosis (TB) are co-endemic and together share a major global burden, with TB alone affecting nearly 10 million individuals annually and helminths infecting over 2 billion people worldwide [1, 2]. Moreover, helminth infections manifest in poor resource settings and overlap geographically with TB infection, especially in lower as well as in some middle-income countries. The helminth larvae travel via the lungs and are known to modulate immunity in the lungs and thus afford a plausible mechanism for helminth infection to influence the immune response against TB [3]. Similarly, TB disease establishes a broad clinical spectrum varying from asymptomatic, latent TB infection to active pulmonary or extra-pulmonary TB disease. However, only 5–10% of the latently infected individuals advance towards pulmonary TB during their lifetime [1]. Cytokines are the crucial players in host resistance to both pulmonary and extra-pulmonary TB [4, 5]. Type 1, Type 17 and other pro-inflammatory cytokines are important for host protection and Type 2 and regulatory cytokines are detrimental for host immunity against active TB [1].

Typically, helminths are eukaryotic microorganisms capable of inducing chronic infections in humans and give rise to morbidity in adults as well as cause physical and cognitive growth deficits in children [6]. Among different helminths, approximately 30 to 100 million people are infected worldwide by *Strongyloides stercoralis* (Ss) parasites [7, 8]. The Ss infected person becomes clinically asymptomatic and has longstanding infection due to the auto infective life cycle and due to the regulation of the host immune system by the parasite [2, 9]. Recent epidemiological and experimental studies have shown that intestinal helminths modulate or alter the host immune response against TB infection and disease [10]. However, their role in regulating the systemic cytokine responses in TBL has not been examined so far.

Therefore, we postulated that the regulatory network established during Ss infection might impact the cytokine profile in TBL disease. To address this, we measured the systemic and TB- antigen stimulated levels of different cytokines of Ss+ and Ss- coinfected individuals. We show TBL-Ss+ individuals have significantly increased bacterial burden in the affected lymph nodes when compared to TBL-Ss- individuals. Our results also show a significantly elevated systemic Type 2, regulatory, Type 17 and reduced pro-inflammatory cytokine response in co-infected helminth-TBL infection. In addition, our results show significantly diminished unstimulated or mycobacterial—antigen stimulated levels of Type 1, Type 17 or IL-1 family cytokines in TBL-Ss+ coinfected individuals.

## Materials and methods

### Ethics

All individuals (participants above 18 years of age were enrolled in the study) were assessed as part of a natural history study protocol approved by Institutional Review Boards of National Institute for Research in Tuberculosis (NIRT, NIRTIEC2010007) and informed written consent was obtained from all participants involved in the study.

### Study population

We studied a group of 88 individuals with TBL, 44 of whom were infected with Ss infection (hereafter Ss+) and 44 of whom were negative for Ss infection and only had TBL (hereafter Ss-) (Table 1). TBL diagnosis was made on the basis of excision biopsy (i.e affected lymph nodes) showing culture positivity for *M*. *tuberculosis*. Culture was performed using homogenized lymph node tissue and culture grades [0 (no colonies)/1+ (20–100 colonies)/2+(>100 colonies)] were used to identify the bacterial burdens as ascertained by growth of *M*. *tuberculosis* on Lowenstein-Jensen solid media [11]. Culture scoring was done independently and blinded to the Ss infection status. Similarly, Ss infection was diagnosed by the presence of IgG antibodies to the 31-kDa recombinant NIE antigen by NIE ELISA. This detection method has been described earlier and found to be the most precise [12, 13]. All the study individuals were BCG vaccinated, negative for HIV and not under any steroid treatment. These individuals are not infected with disseminated strongyloidiasis. We have screened all these individuals for lymphatic filariasis and all of them were negative for filarial infection (based on TropBio ELISA results). We did not perform any stool/microscopy examination for these individuals and they were anti-tuberculosis and anthelmintic treatment naive. All these individuals were from a peri-urban area in Chennai, India.

**Table 1:** Demographics of the study population.

Study Demographics	TBL-Ss+	TBL-Ss-	P Value
Number of subjects recruited (n)	44	44	NS
Gender (M/F)	12/32	14/30	NS
Median age in years (range)	23 (18–53)	25 (19–59)	NS
Culture grade (0/1+/2+)	7/30/7	15/28/1	0.028 ^a^
NIE antibody titres	484.77 (291–1202)	174.3 (38–281.75)	<0.0001^b^

^a^Calculated using the Chi-square test; NS = Not significant

^b^Calculated using the Mann-Whitney test.

### Collection of plasma

Blood samples (10 ml) were collected in sodium heparin tubes and plasma was collected by centrifugation at 2600 revolutions per minute (rpm) for 10 minutes at 4°C and stored at -80°C.

### Measurement of cytokines by Bio-plex ELISA

Circulating plasma cytokine levels were measured using a bio-plex multiplex cytokine assay system (Bio-Rad, Hercules, CA). The analytes measured were IFN-γ, TNF-α, IL-2, IL-4, IL-5, IL-13, IL-12, IL-17A, IL-22, IL-10, IL-1α, IL-1β, IL-6 and GM-CSF. The experiment was carried out according to the manufacturer’s instruction provided (R & D systems). The standard ranges of each cytokine were as follows: IFN-γ-49.18–11,950 pg/ml, TNF-α-8.89–2,160 pg/ml, IL-2-31.11–7,560 pg/ml, IL-4-15.72–3,820 pg/ml, IL-5-6.5–1,580 pg/ml, IL-13-367.78–89,370 pg/ml, IL-12-133.58–32,460 pg/ml, IL-17A-12.47–3,030 pg/ml, IL-22-14.28–3,470 pg/ml, IL-10-3.62–880 pg/ml, IL-1α-5.31–1,290 pg/ml, IL-1β-16.3–3,960 pg/ml, IL-6-4.53–1,100 pg/ml and GM-CSF-11.58–2,900 pg/ml.

### QuantiFERON supernatant ELISA

1ml of heparinized whole blood obtained from a subset of individuals (n = 30 from each group) was incubated with either no antigen (baseline/NIL/negative control) or cocktail of TB peptide antigens (ESAT-6, CFP-10, TB 7.7) or mitogen (phytohaemoggultin-p) for 18 hours, according to the manufacturer’s instructions using the QuantiFERON Gold in-tube kit. The NIL or TB antigen or mitogen stimulated whole blood supernatants were then used to measure the levels of IFN-γ, TNF-α, IL-17A, IL-22, IL-1α and IL-1β using the Duo-set ELISA kits from R& D systems. The net cytokine values for TB antigen or mitogen stimulation was calculated by subtracting the baseline (NIL) values for each individual.

### Statistical analysis

All the data were analyzed using GraphPad PRISM (GraphPad Software, Inc., San Diego, CA, USA) tool. Geometric means (GM) were used for measurements of central tendency and nonparametric Chi-square test was used to compare statistically significant differences in age, gender and bacterial burdens. Mann-Whitney U test was used to compare the statistically significant differences among the cytokines analyzed.

## Results

### Study population demographics

The demographics of the study population are listed in Table 1. There were no significant differences in age and gender between TBL-Ss+ and TBL-Ss- individuals. However, TBL-Ss+ individuals had significantly higher bacterial burdens as determined by the culture grade in solid media of the affected lymph node. Table 2 represents the various hematological parameters of the study population. Among them, absolute lymphocyte and monocyte counts were significantly decreased and absolute neutrophil and eosinophil counts were significantly elevated in TBL-Ss+ when compared to TBL-Ss- individuals.

**Table 2:** Hematological profile of the study population.

Hematological profile	TBL-Ss+	TBL-Ss-	p value^a^
Whole blood cells (10^3^/μL)	6.01 (4.9–10.9)	6.36 (3.80–11.9)	NS
Red blood cells (10^6^/μL)	3.98 (3.94–8.9)	4.14 (3.92–5.44)	NS
Lymphocyte count (cells/μL)	1.76 (0.88–3.59)	2.03 (0.87–4.27)	**0.0476**
Monocyte count (cells/μL)	436.37 (102.9–2037.7)	501.33 (213.0–1331.2)	**0.0380**
Eosinophil count (cells/μL)	223.08 (50.4–723.6)	207.48 (44.1–1570.8)	**0.0322**
Basophil count (cells/μL)	65.77 (17.4–703.5)	61.78 (7.2–2.93.8)	NS
Neutrophil count (cells/μL)	3729.6 (2245.6–7644.0)	3562.4 (2263.5–7819.6)	**0.0298**
Platelet count (cells/μL)	19478.8 (11799.0–52538.0)	20656.4 (6444.0–67362.0)	NS
Hemoglobin (g/dl)	9.7 (8.0–18.3)	11.02 (8.0–14.8)	NS

^a^Calculated using the Mann-Whitney test; NS = Not significant.

### TBL-Ss+ individuals exhibit significantly elevated levels of Type 2, Type 17 and regulatory cytokines but not Type 1 and other pro-inflammatory cytokines

**Fig 1:**
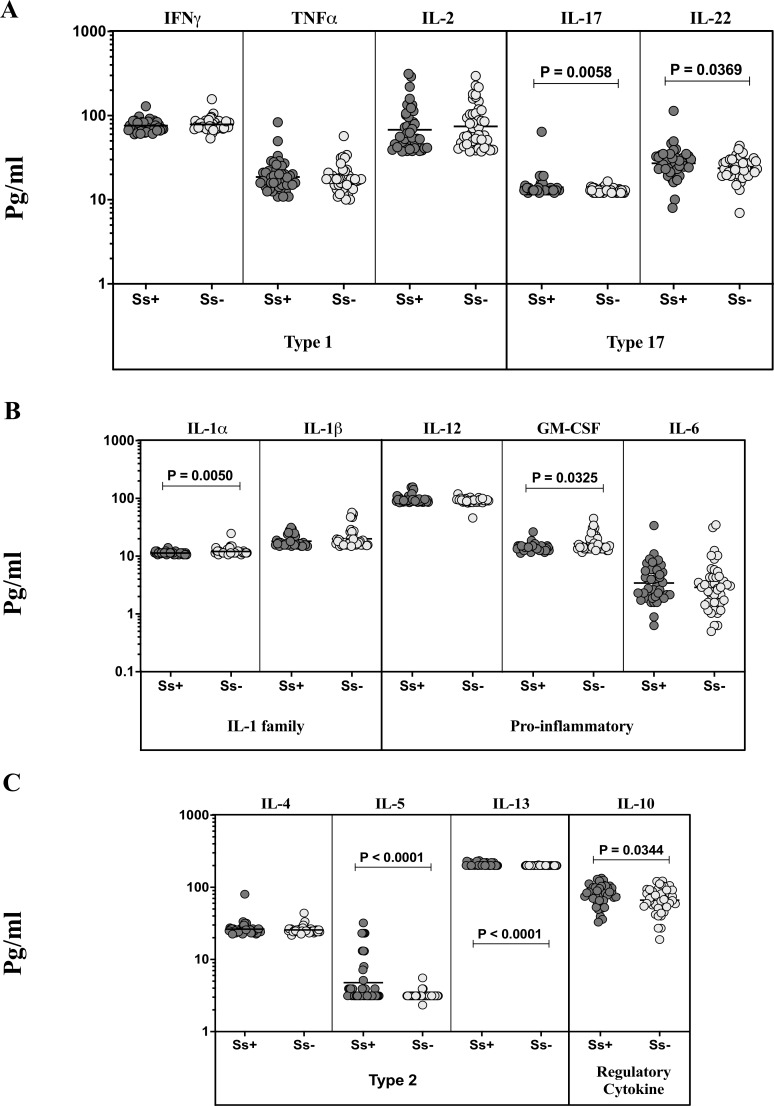
TBL-Ss+ individuals exhibit elevated plasma levels of Type 17, Type 2 and diminished levels of pro-inflammatory cytokines. (A) The plasma levels of Type 1 (IFN-γ, TNF-α and IL-2), Type 17 (IL-17 and IL-22), (B) pro-inflammatory (IL-1α, IL-1β, IL-12, GM-CSF and IL-6), (C) Type 2 cytokines (IL-4, IL-5 and IL-13) and regulatory (IL-10) cytokines were examined by multiplex ELISA in TBL individuals with *Strongyloides stercoralis* (Ss) (Ss+, n = 44) or without Ss coinfection (Ss-, n = 44). The results are represented as scatterplots with each circle representing a single individual and the bar representing the geometric mean (GM). P values were calculated using the Mann-Whitney U test.

To assess the impact of coincident Ss infection on Type 1, Type 2, Type 17, regulatory and pro-inflammatory cytokines, we measured the plasma levels of Type 1 (IFN-γ, TNF-α, IL-2), Type 2 (IL-4, IL-5, and IL-13), regulatory (IL-10), Type 17 (IL-17A and IL-22) and other pro-inflammatory (IL-1α, IL-1β, IL-6, IL-12) cytokines in TBL-Ss+ and TBL-Ss- individuals (Fig 1). As shown in Fig 1A, the circulating levels of Type 1 [IFN-γ (Geometric mean (GM) of 75.89 pg/ml vs 78.53 pg/ml, p = 0.2756), TNF-α (GM of 18.64 pg/ml vs 17.6 pg/ml, p = 0.6045) and IL-2 (GM of 67.84 pg/ml vs 70.56 pg/ml, p = 0.3657)] cytokines were not significantly different between TBL-Ss+ and TBL-Ss- individuals. However, both the Type 17 cytokines—IL-17A (GM of 14.04 pg/ml vs 10.91 pg/ml, p = 0.0058) and IL-22 (GM of 27.16 pg/ml vs 21.75 pg/ml, p = 0.0369) were significantly elevated in TBL-Ss+ individuals in comparison to TBL-Ss- individuals. As shown in Fig 1B, the circulating plasma levels of IL-1α (GM of 10.37 pg/ml vs 12.04 pg/ml, p = 0.0050) and GM-CSF (GM of 12.17 pg/ml vs 16.51 pg/ml, p = 0.0325) but not the other pro-inflammatory cytokines were significantly diminished in TBL-Ss+ individuals when compared to TBL-Ss- individuals. Finally, as shown in Fig 1C, the circulating levels of IL-5 (GM of 4.772 pg/ml vs 3.189 pg/ml, p<0.0001), IL-13 (GM of 207.4 pg/ml vs 190.9 pg/ml, p<0.0001) and IL-10 (GM of 78.97 pg/ml vs 66.43 pg/ml, p<0.0344) but not IL-4 were significantly elevated in TBL-Ss+ individuals in comparison to TBL-Ss- individuals. Hence, TBL-Ss coinfection is characterized by altered circulating levels of different cytokines.

### TBL-Ss+ individuals exhibit significantly diminished unstimulated and TB-antigen stimulated levels of Type 1, Type 17 and IL-1 family of cytokines

**Fig 2:**
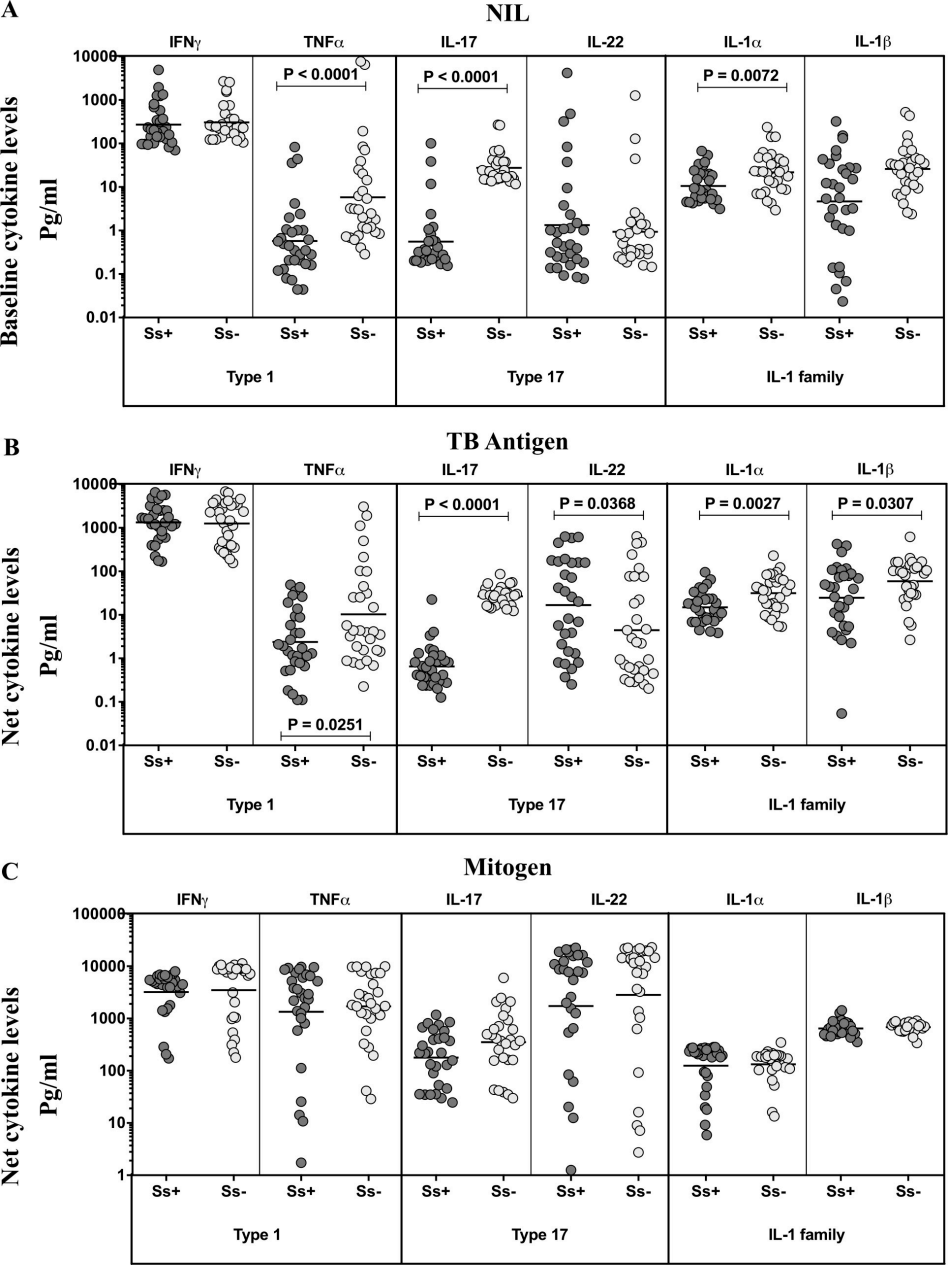
TBL-Ss+ individuals exhibit diminished unstimulated and mycobacterial antigen stimulated levels of Type 1 and Type 17 cytokines. (A) The baseline/NIL (no antigen/negative control) or (B) mycobacterial antigen-stimulated or (C) mitogen stimulated levels of IFN-γ, TNF-α, IL-17, IL-22, IL-1α and IL-1β were examined by multiplex ELISA in Ss+ and Ss- individuals. The results are shown as scatterplots with each circle representing a single individual and the bar representing the GM. P values were calculated using the Mann-Whitney U test.

To assess the influence of Ss co-infection on TB antigen stimulated cytokine levels, we measured the systemic levels of these cytokines upon stimulation of whole blood with no antigen (Nil) or with TB peptide antigens (ESAT-6, CFP-10, TB 7.7) or mitogen in a subset (n = 30 from each group) of TBL-Ss+ and TBL-Ss- individuals (Fig 2). As shown in Fig 2A, the unstimulated levels of TNF-α (GM of 1.5734 pg/ml vs 5.798 pg/ml), IL-17 (GM of 2.5577 pg/ml vs 27.49 pg/ml), and IL-1α (GM of 10.57 pg/ml vs 21.81 pg/ml) but not IFN-γ, IL-22 and IL-1β were significantly diminished in TBL-Ss+ compared to TBL-Ss- individuals. Similarly, as shown in Fig 2B, the TB-antigen stimulated levels of TNF-α (GM of 2.077 pg/ml vs 10.31 pg/ml), IL-17 (GM of 2.6532 pg/ml vs 26.74 pg/ml), IL-1α (GM of 14.93 pg/ml vs 31.58 pg/ml) and IL-1β (GM of 24.72 pg/ml vs 59.52 pg/ml) were also significantly decreased in TBL-Ss+ compared to TBL Ss- mono infected individuals. Finally, upon mitogen stimulation, no significant differences were observed among the cytokines measured between the study groups. Thus, TBL Ss+ is associated with diminished levels of unstimulated and TB antigen stimulated Type 1, Type 17 and IL-1 family cytokines.

### PCA analysis of cytokines did not distinguish TBL Ss+ and TBL Ss- individuals

**Fig 3:**
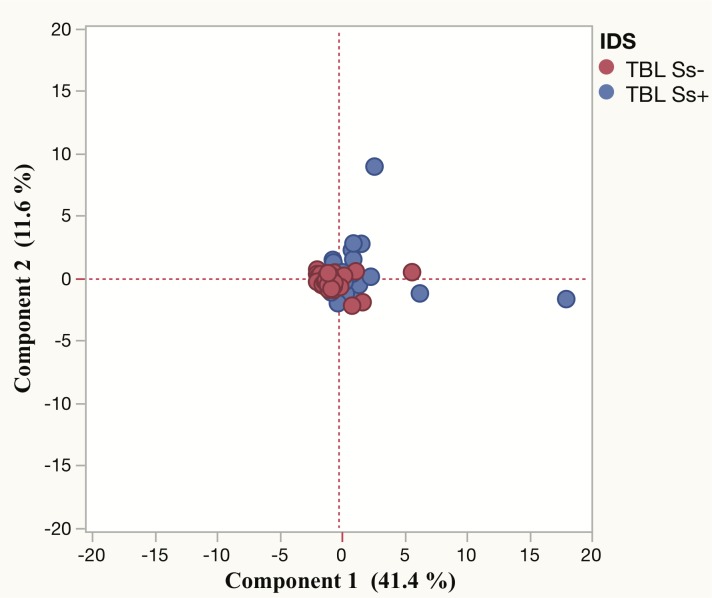
Principle component analysis (PCA) plots of cytokine values in TBL Ss+ and TBL Ss- individuals. PCA plot shows the cytokine ELISA data from the combination of two different experimental groups TBL Ss+ (blue circles) and TBL Ss- (red circles). The PCA represents the two principal components of variation.

Next, to examine the impact of all the plasma cytokines measured, we used the cytokine data to generate the PCA models between the TBL Ss+ and Ss- individuals. As shown in Fig 3, we did not observe any distinct clustering of cytokines analyzed using PCA analysis between the two-study populations.

## Discussion

Extra-pulmonary TB accounts for ~20% of all TB cases and lymph nodes are often the most common site. Diagnosis of TBL is challenging due to the paucibacillary nature of the disease and therefore, the pathogenesis of this disease is not well understood. Previous studies have shown that helminth infections can influence the pathogenesis and immune response to pulmonary TB [10, 14]. Also, infection with helminths induces a regulatory response to suppress anti-helminth and other pathological reactions at numerous levels and to modulate the host immunity to other chronic infections as well [15–17]. Our previous study described that co-infected Ss infection with active or latent TB has significantly reduced Type 1 (IFN-γ, TNF-α, and IL-2), Type 17 (IL-17A and IL-17F) and increased regulatory as well as Type 2 (IL-4, IL-5, and IL-13) cytokines when compared to TB infection alone [14]. In addition, both filarial and hookworm infections also potentially modulate Th1 and Th17 cytokines in an antigen-specific manner [18, 19]. We expand our data on those findings and report the influence of Ss coinfection on the circulating and antigen stimulated cytokines in TBL disease. We also report for the first time (to our knowledge) that co-infected helminth infection is associated with higher bacterial burdens in the affected lymph node of TBL individuals. This needs to be validated in a larger cohort but has profound implications in terms of TBL pathogenesis and the clinical management of TBL.

Helminth infections are typically associated with Type 2 cytokine responses with elevated levels of IL-4, IL-5 and IL-13 [2, 20, 21]. Similarly, they are also associated with the induction of regulatory cytokines, including IL-10 [22, 23]. We have previously shown that this pattern is true for Ss infection as well [14]. Our study reveals that in the presence of Ss infection, TBL is also characterized by a significant (albeit moderate) enhancement of Type 2 and regulatory cytokines. This suggests a potential influence of Ss coinfection on host protective autophagy, antimicrobial activity, Type 1 cytokine and other intracellular protective immune responses [24]. Therefore, there might be a higher risk of dissemination and expansion of TBL disease in Ss coinfected individuals. Elevated levels of IL-10 has a potential role in modulating the immune responses to TB infection/ disease [25]. In contrast, no significant association was found for Type 1 cytokines (IFN-γ, TNF-α, and IL-2) which are usually associated with the defensive function in both latent and pulmonary TB [1, 26]. This might be due to TBL being a paucibacillary infection. Moreover, we have previously shown in TBL, Type 1 cytokines were not significantly associated with any modulation when compared with latent TB individuals [27]. In addition, it was previously reported Type 1 cytokines were significantly reduced and IL-4 were significantly enhanced in both LTB and active TB co-infected with Ss infection [14]. IL-17 also plays a protective role against TB by regulating the cytokine (IL-12 and IFN-γ) balance thereby inhibiting the disease pathogenesis inside the host [28]. In contrast, IL-17 might also influence the immuno-pathogenesis with multidrug-resistant TB disease and promote severe tissue damage [29]. Meanwhile, the role of IL-22 in TB infection remains poorly understood. Both IL-17 and IL-22 were earlier shown to be downmodulated in latent and active TB cases [14], which is in contrast to our findings in plasma. Therefore, TBL appears to exhibit a different cytokine profile in the context of helminth coinfection in comparison to active or latent TB. Thus, TBL might be placed in the middle of the spectrum between active and latent TB in the field of clinical TB.

Since plasma cytokines reflect the global cytokine milieu and can be modulated by a variety of confounding factors, we have measured the antigen specific cytokines using the QuantiFERON supernatants. Both baseline and TB antigen stimulated supernatant levels of TNF-α but not IFN-γ were significantly downregulated in Ss- co-infected individuals. It has been reported that TB antigen-specific type 1 cytokines are normally involved with protective immune response in latent TB infection [1]. Likewise, both baseline and antigen-specific IL-17A responses were diminished and IL-22 antigen-specific responses were elevated with Ss coinfected individuals. IL-17A has been associated with protective immunity to TB disease in mice whereas the function of IL-22 in is poorly defined [30]. Therefore, our data suggests that ongoing helminth co-infection could potentially alters the course of TBL disease. We also studied a panel of pro-inflammatory cytokines, including IL-1α, IL-1β, IL-12, GM-CSF, and IL-6 in both Ss+ and Ss- individuals. IL-1α and GM-CSF are cytokines involved in the protective immune responses against TB in animal models. Interestingly, both these cytokines were down-modulated by Ss coinfection. This data suggests that certain pro-inflammatory cytokines are depressed by Ss infection [14] and this might reflect dampened inflammation in TBL and/or an effect on the host protective immunity as well. To support the data, both the unstimulated and TB antigen-stimulated levels of IL-1 family cytokines were also altered in Ss+ co-infected individuals. This might be additional evidence that helminth coinfection can modify the protective immunity against TB infection. Interestingly, Ss infection appears not to exert any cell intrinsic effect on the ability to produce cytokines since both TBL-Ss+ and TBL-Ss- individuals did not exhibit any differences in mitogen induced cytokine production.

We have also shown that coinfected individuals were associated with reduced lymphocytes, monocytes and elevated neutrophils and eosinophils in comparison with TBL-Ss- individuals. We have shown that helminth coinfection can potentially skew the immune response in TBL disease and potentially modify the immunity to TB antigens as well. This effect could have implications in terms of designing better therapeutic drugs as well as vaccines for TBL [31]. Our data on PCA analysis suggests that the combined analysis of all the cytokines analyzed is not sufficient to cluster segregate the groups and more refined analysis of specific cytokines might be needed. Finally, Ss coinfected individuals did not exhibit any significant difference in the plasma levels of CC or CXC chemokines in comparison to Ss- individuals. Overall, these findings will be useful to understand the host protective immunity against TB-helminth coinfections in co-endemic settings. In addition, elimination of helminth co-infection among TBL individuals might improve the efficacy of the immune responses to TBL disease.
